# An interpretable radiomics model based on contrast‑enhanced pancreatic computed tomography for predicting the prognosis of post-acute pancreatitis diabetes mellitus

**DOI:** 10.1186/s12880-026-02258-7

**Published:** 2026-03-13

**Authors:** Ran Hu, Yan-Li Chen, Gang-Jing Li, Yin-Deng Luo, Di Zhou, Zhi-Gang Wang, Xiao-Di Zhang, Zhixuan Song, Wei Chen, Hua Yang

**Affiliations:** 1 Department of Radiology, Chongqing Hospital of Traditional Chinese Medicine (The First Affiliated Hospital of Chongqing University of Chinese Medicine), No.6, Panxi 7th Road, Jiangbei District, Chongqing, 400021 PR China; 2 Department of Radiology, Southwest Hospital, Army Medical University (Third Military Medical University), 30# Gaotanyan Road, Shapingba District, Chongqing, 400038 China; 3 Department of Radiology, The Second Affiliated Hospital of Chongqing Medical University, 76 Linjiang Road, Yuzhong Distinct, Chongqing, 400010 PR China; 4 Department of Radiology, The First Affiliated Hospital of Chongqing Medical University, No.1, Yuanjiagangyouyi Road, Yuzhong District, Chongqing, 400010 PR China; 5 Department of Ultrasound, The Second Affiliated Hospital of Chongqing Medical University, 76 Linjiang Road, Yuzhong Distinct, Chongqing, 400010 PR China; 6 The Department of Clinical Science, Philips Healthcare, Chengdu, Sichuan China; 7 Clinical and Technical Support, Philips Healthcare, Guangzhou, China

**Keywords:** Radiomics, X-ray computed tomography, Acute pancreatitis, Diabetes mellitus, Complications

## Abstract

**Purpose:**

To develop a radiomics model utilizing contrast-enhanced computed tomography (CECT) for predicting the prognosis of post-acute pancreatitis diabetes mellitus (PPDM-A) and to explain the model’s internal predictive mechanisms using Shapley Additive exPlanations (SHAP).

**Methods:**

226 PPDM-A patients were retrospectively recruited from three centers, with 107 in training, 46 in internal, and 73 in external cohorts. There were 34, 15 and 28 patients with complications in each cohort. The complications included microvascular complications, infection, diabetic ketosis and hypoglycemia. In PPDM-A patients’ first pancreatitis episode, 2398 radiomics features were extracted from CECT images (arterial and venous phases). FeAture Explorer generated the machine learning pipeline and selected important radiomics features. Gaussian processes classifiers built radiomics and clinical-radiomics models, while Naive Bayes classifiers built the clinical model. The SHAP method was applied to provide insights into the model’s predictive process.

**Results:**

The radiomics model predicted PPDM-A complications with the area under the receiver operating characteristic curve (AUC) of 0.95, 0.888, and 0.948 in training, internal, and external cohorts. In external cohort, the radiomics model significantly outperformed the clinical model (AUC 0.948 vs. 0.713, *p* = 0.002), while the combined model showed no significant difference from the radiomics model (AUC 0.933 vs. 0.948, *p* = 0.638). The SHAP technology provided physicians with insights into the global and individual impacts of radiomic features on model predictions.

**Conclusion:**

The CECT‑based radiomics model showed favorable prognostic performance for the prognosis of PPDM‑A. SHAP analysis interpreted the model’s mechanism, enhancing its clinical reliability and transparency.

**Supplementary Information:**

The online version contains supplementary material available at 10.1186/s12880-026-02258-7.

## Introduction

Post-acute pancreatitis diabetes mellitus (PPDM-A) is a common long-term sequela of acute pancreatitis (AP) [1, 2]. Evidence increasingly shows that even mild AP is not self-resolving, as it significantly raises the risk of metabolic complications [3]. Recent studies suggest that 23% of AP patients develop diabetes, with mild AP patients facing more than double the diabetes risk compared to the general population [4, 5]. The prevalence of PPDM-A has almost tripled over the past decade, and its incidence is projected to increase by 2.8% annually, reaching 15.8 per 100,000 population by 2050 [6], making it a growing global health concern.

A lack of awareness often misclassifies PPDM-A as type 2 diabetes mellitus (T2DM). Compared to T2DM, PPDM-A patients have poorer glycemic control, higher insulin dependence after diagnosis [7], and a significantly elevated risk of severe complications [8]. Additionally, PPDM-A is strongly linked to higher mortality from pancreatic cancer, infectious diseases, and gastrointestinal disorders [9, 10], with young and middle-aged patients experiencing shorter life expectancy [11]. These adverse outcomes highlight the critical need for early prediction of PPDM-A and its complications to guide targeted interventions and improve patient prognosis.

Previous research primarily concentrated on identifying risk factors and clinical outcomes associated with PPDM-A, but stable and precise predictive models for PPDM-A complications were lacking [7–15]. Radiomics offers a solution by automatically analyzing extensive quantitative imaging data with high throughput and leveraging machine learning techniques to extract features for disease diagnosis and prediction [16]. Our prior study developed a radiomics model for predicting PPDM-A occurrence, achieving an accuracy of 0.968 in external validation [17]. Another study also supports the application of radiomics, highlighting radiomic features’ ability to detect pancreatic changes [18]. However, these studies only address the “risk of developing PPDM-A” rather than the “prognosis of PPDM-A patients,” leaving a critical gap in predicting complications that directly impact patient survival and quality of life.

Moreover, physicians remain cautious about adopting radiomics models due to their unclear internal mechanisms and unexplained features—a “black box” problem that hinders clinical translation [19]. The Shapley Additive exPlanations (SHAP) method addresses this issue by integrating six interpretation methods into a unified framework, defining feature importance and providing theoretical results [20]. SHAP highlights feature significance and their influence on predictions, elucidating the importance of individual features to the model’s output [21]. By combining SHAP with radiomics, we can present models in a more transparent and understandable manner [22].

Building on our previous work, this study advances the field by focusing on PPDM-A prognosis rather than just incidence. We developed a radiomics model using arterial- and venous-phase contrast-enhanced computed tomography (CECT) images from three tertiary centers. By integrating the SHAP method, this study has improved the model’s interpretability, enabling clinicians to clearly understand the model’s predictive mechanisms. This study is expected to provide a non-invasive, accurate, and interpretable reference tool for predicting PPDM-A complications in clinical practice, offering certain practical basis for personalized risk stratification and early clinical intervention. Therefore, we analyzed PPDM-A complications and developed a radiomics model using CECT images of patients at their initial AP onset to predict the prognosis of PPDM-A. Furthermore, we integrated the radiomics model with SHAP to provide explanations and visualizations for our model.

## Materials and methods

### Patients

This retrospective, multicentric study adhered to the Helsinki Declaration (2013) and received ethical approval from the institutional review boards of the First Affiliated Hospital of Chongqing Medical University (institutions 1), the Second Affiliated Hospital of Chongqing Medical University (institutions 2) and Southwest Hospital (institutions 3), (approval number: 315, Dec. 30, 2021, retrospectively registered). Due to the retrospective design, patient informed consent was waived. We enrolled patients with AP from three hospitals between January 2014 to August 2022, collected and analyzed their baseline information and imaging data.

The diagnostic criteria for acute pancreatitis and diabetes are shown in Section S1 in the Supplementary Material.

The inclusion and exclusion criteria for PPDM-A patients are: inclusion criteria: (a) Diabetes diagnosed > 90 days after initial AP [1]. (b) Upper abdominal CECT within 7 days of AP onset. (c) Inpatients. Exclusion criteria: (a) Prior history of diabetes. (b) Elevated blood glucose during hospitalization. (c) High blood glucose within 90 days after discharge. (d) Acute exacerbations of chronic pancreatitis. (e) Cancer or severe chronic debilitating conditions. (f) Loss to follow-up or inadequate image/medical records. (g) Age < 18 years.

PPDM-A complications was diagnosed by recording at least one of the following [23–25]: (a) diabetic ketoacidosis; (b) hypoglycemia; (c) microvascular complications (nephropathy, neuropathy, retinopathy, diabetic foot) or (d) infections (pneumonia, abdominal, urinary tract). Diagnostic criteria for these conditions are detailed in Section S2 of the Supplementary Material.

Through medical records and/or telephone interviews, we identified patients with first-onset AP between January 2014 and August 2022 who subsequently developed diabetes. Patients diagnosed with PPDM-A were enrolled, while those who did not develop diabetes were excluded. Enrolled PPDM-A patients were further followed up for complications and stratified into a complication group and a non-complication group. The non-complication group was monitored until August 2024 with no complications recorded. In the complications group, the average time between the initial AP episode and complications diagnosis was 90.26 ± 28.24 months (32–147 months). For the non-complications group, the average follow-up was 86.89 ± 31.08 months (25–149 months).

PPDM-A patients from institutions 1 and 2 were randomly assigned to the training and internal validation cohorts, while those from institution 3 formed the external validation cohort.

**Fig. 1:**
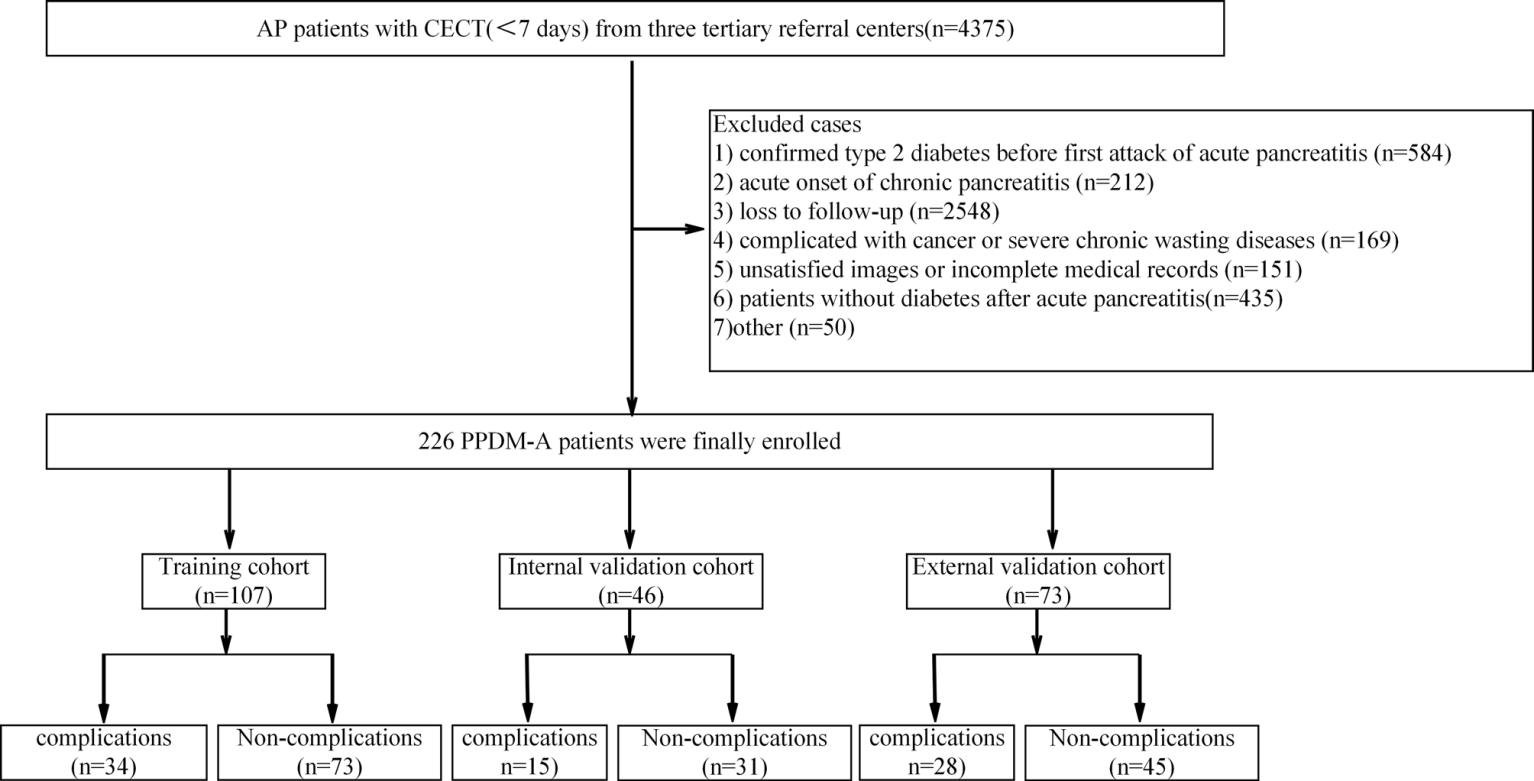
The flowchart of patient selection and study design. PPDM-A, post-acute pancreatitis diabetes mellitus

Twenty-eight clinical characteristics were collected, including sex, age, fatty liver, recurrence, etiology, follow-up duration, hospital stay, alcohol/smoking habits, pancreatic necrosis, CT severity index (CTSI), the 2012 Revised Atlanta Criteria, extra-pancreatic inflammation on CT (EPIC), Triglyceride acid, Cholesterol, Ca+, leukocyte, hemoglobin, Total protein, Albumin, Total bilirubin, Direct bilirubin, Urea, Creatinine, Alanine Transaminase (ALT), Aspartate Transaminase (AST), Gamma-Glutamyl transferase (GGT), Alkaline Phosphatase (ALP). The patient selection and study design flowchart shown in Fig. 1.

### CECT image collection, image segmentation, preprocessing, radiomics feature extraction and reproducibility evaluation

#### CECT image collection

All patients underwent an abdominal CECT examination within 7 days following the onset of AP symptoms using one of six multidetector row CT systems. Anonymous CECT images from both the arterial and venous phases were retrieved from the picture archiving and communication system for the purpose of feature extraction. The detailed image collection information is provided in Section S3 in the Supplementary Material.

#### Image segmentation, preprocessing, and radiomics feature extraction

Two abdominal radiologists, with 8 and 12 years of experience respectively, independently utilized the IntelliSpace Discovery platform (ISD) from Philips Healthcare (Best, the Netherlands) without any knowledge of the patients’ clinical outcomes. On the ISD platform, a semi-automatic process was employed to generate a three-dimensional region of interest (3DROI) encompassing the entire pancreas by delineating the pancreatic boundaries on the CECT images. This process included pancreatic necrosis while excluding the bile ducts and blood vessels. Subsequently, the pyradiomics 2.1 plugin on the ISD platform was utilized to extract radiomic features from each 3DROI. A total of 1,199 radiomic features was extracted, including shape-based features, first-order histogram features, high-order textural features, and transformed features. For each patient, two distinct 3DROIs from the CECT images of both the arterial and portal venous phases were used, and all 2,398 radiomic features from these two 3DROIs were integrated.

To ensure the reliability and reproducibility of the study results, we conducted rigorous preprocessing of the data and images. On one hand, since six different CT scanners were employed for image acquisition in this study, and these scanners were configured with distinct acquisition techniques and scanning parameters, we performed resampling on the images prior to feature extraction to minimize the impact of CT parameters on radiomics features. The voxel resolution of all images was uniformly adjusted to 1 mm × 1 mm × 1 mm. On the other hand, different radiomics features exhibit distinct value ranges, which hinders direct comparisons between features of varying orders of magnitude. Therefore, prior to data analysis, we adopted the Z-score normalization method for data processing. Also referred to as zero-mean normalization, this method performs transformation based on the mean and standard deviation of the dataset. It converts the original data into a distribution with a mean of 0 and a standard deviation of 1. The specific formula is as follows:

$$X^{\prime}=\frac{X-\mu}{\sigma},$$

where denotes the normalized data, represents the original data, is the mean of the dataset, and is the standard deviation of the dataset.

#### Radiomics features reproducibility evaluation

**Fig. 2:**
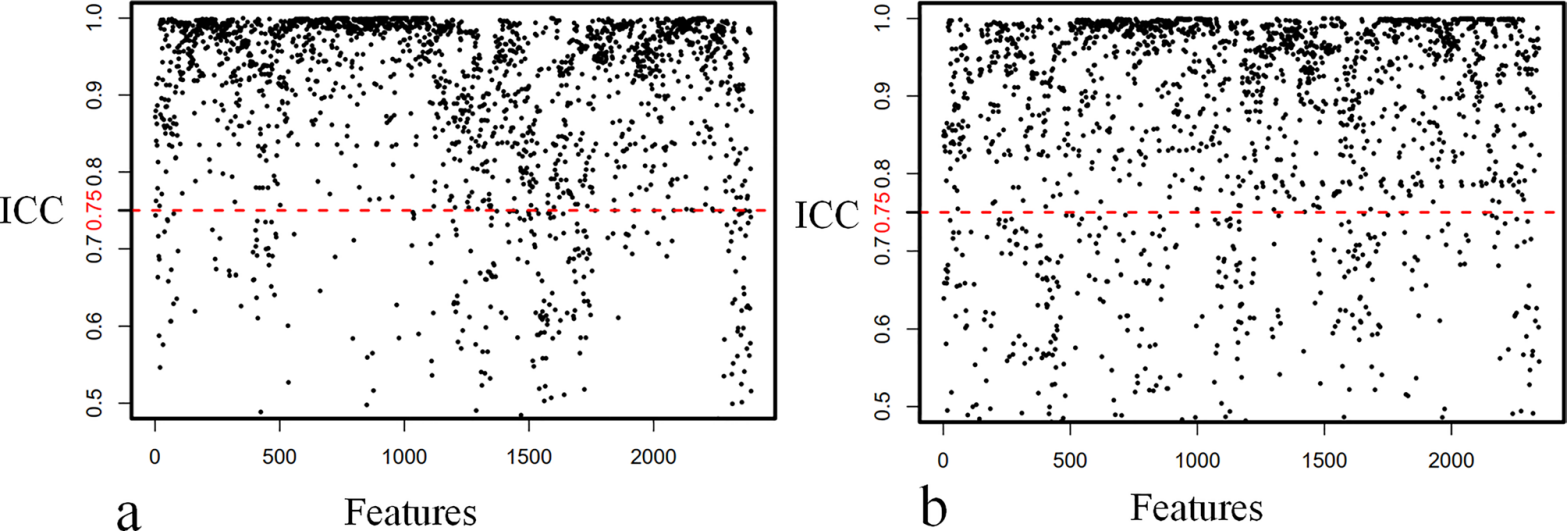
Radiomics features reproducibility evaluation (**a**) Features demonstrated strong intra-observer reliability with Intraclass correlation coefficients (ICC) scores exceeding 0.75 (marked by the red threshold line). (**b**) Similarly, robust interobserver agreement was observed for all features, with ICC values also surpassing 0.75

Two abdominal radiologists, blinded to clinical outcomes, evaluated CECT images of 30 patients to assess feature extraction consistency. For intra-observer reliability, the first observer delineated 3DROIs and extracted features twice within 7 days. For inter-observer agreement, the second observer delineated 3DROIs once and compared features with the first observer. Intraclass correlation coefficients (ICCs) were calculated to quantify reliability and agreement. Features with ICC > 0.75 were retained. Intra-observer reliability showed 2106 features with an average ICC of 0.972, excluding 292. Inter-observer reliability showed 1815 features with an average ICC of 0.967, excluding 583. Notably, 178 features were commonly excluded in both intra- and inter-observer assessments. Ultimately, 697 features were omitted, leaving 1701 features for further analysis. The details illustrated in Fig. 2.

### Radiomics feature selection, model construction and validation

FeAture Explorer (FAE, version 0.5.13) was used to perform machine learning modeling exploration based on features extracted by IntelliSpace Discovery platform (Philips Healthcare, Best, the Netherlands) for developing and comparing radiomics models. It offered various data processing and model building methods and formed various machine learning pipelines consisting of steps such as feature normalization, feature selection, and classification. Then, an optimal pipeline with appropriate combinations and important radiomics features were selected by comparing combinations of different methods. During the feature preprocessing, data were randomly divided into training and internal validation cohorts at a proportion of 7:3. We applied normalization to the feature matrix and reduced the dimension of the feature space with Pearson correlation coefficients (PCC). If the PCC exceeded 0.9, one of the features was randomly removed. Before building the radiomics model, we used the analysis of variance (ANOVA) algorithm, Relief, recursive feature elimination (RFE), and the Kruskal-Wallis test to select features. We considered the number of features ranging from 1 to 10. Then, we used ten machine learning classifiers to build the radiomics models, which including support vector machines, linear discriminant analysis, AdaBoost, Gaussian processes, autoencoders, random forests, logistic regression, lasso logistic regression, decision trees, and Naive Bayes. Lastly, we utilized the 5-fold cross-validation method to assess the results. Additionally, the performance metrics such as accuracy, sensitivity, specificity, positive predictive value and negative predictive value were computed at a cutoff value that maximized the Youden index for each predictive model. The area under the receiver operating characteristic curve (AUC) was then calculated for each tested condition.

This study integrated four feature selection methods, set the number of features within a range of 1 to 10, and adopted ten distinct classifiers, ultimately constructing 400 machine learning models. These models were ranked in descending order based on their AUC values from the training and internal validation cohorts. After excluding overfitted models, the optimal model with the highest AUC value was identified, which employed ANOVA as the feature selection method, included 6 features, and used Gaussian Processes as the classifier.

### Clinical and combined (clinical-radiomics) model construction

The clinical model construction was based on clinical features from the training cohort adopting the FAE software. Feature selection and dimension reduction are based on the method of ANOVA, Relief, RFE, the Kruskal-Wallis test and PCC, respectively. Naive Bayes classifier was used to select the most optimal features of model-building. The performance of the clinical model was tested in the validation cohort.

The combined model construction was based on the final selected radiomics (6 features) and the whole of clinical features from the training cohort adopting the FAE software. Each feature is normalized by using Z-score technique. Feature selection and dimension reduction are based on the method of ANOVA, Relief, RFE, the Kruskal-Wallis test and PCC, respectively. Gaussian processes classifier was used to select the most optimal features of model-building. The performance of the combined model was tested in the validation cohort.

### Model interpretability with SHAP

The SHAP method was utilized to interpret and comprehend the radiomic features incorporated within the radiomic models. It serves as a tool to visualize the significance of each feature in the intricate machine learning model and elucidate how each feature within the model affects the probability of a specific output, either by increasing or decreasing it.

**Fig. 3:**
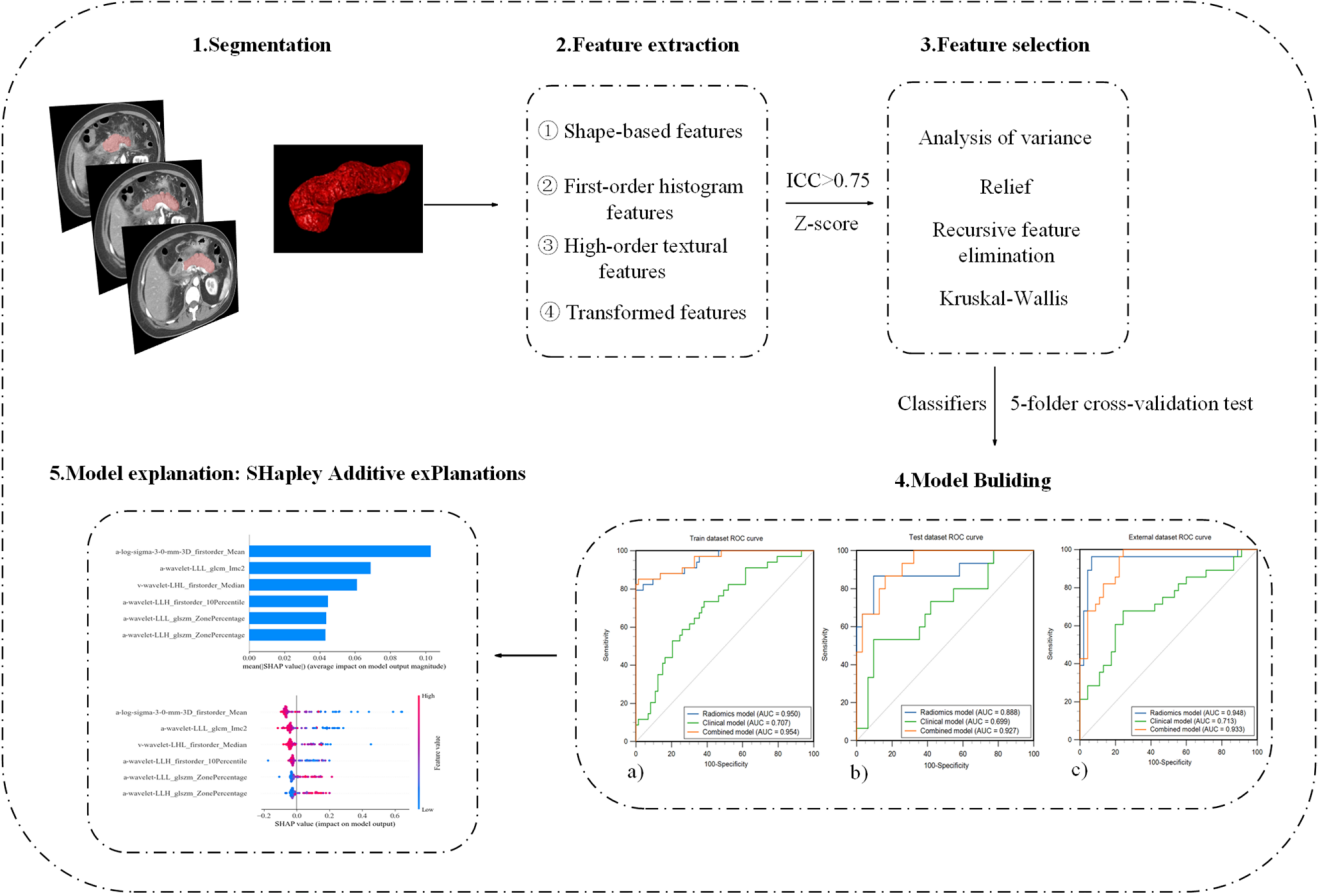
The workflow in this study

The workflow is presented in Fig. 3.

### Statistical analysis

Statistical analyses were conducted using SPSS for Windows (version 22.0, Chicago, IL, USA). Data normality was checked by the Shapiro–Wilk test. Normally distributed variables were reported as mean ± standard, skewed ones as median (Inter-Quartile Range). Continuous variables were compared using the independent-sample t test or the Mann–Whitney U test. Categorical variables were tested using Chi-squared or Fisher’s exact tests. DeLong test was used to compare AUCs of the models. Statistical significance was set at *P* < 0.05 (2-sided).

## Results

### Patient characteristics

A total of 226 patients with PPDM-A (mean age: 46 ± 14 years, range: 18–96 years, 168 males) was enrolled, including 77 with PPDM-A complications and 149 without. The complication rates across the training, internal validation, and external validation cohorts were 31.78%, 32.61%, and 38.36%, respectively, with no statistically significant difference (*p* = 0.64). Among the 28 clinical characteristics, only triglyceride levels differed significantly between the complications and non-complications groups across all cohorts (*p* = 0.036, 0.01, 0.001). Additionally, patients with complications were younger in the training and external validation cohorts (*p* = 0.032, 0.01), and had higher cholesterol levels in the internal and external validation cohorts (*p* = 0.02, 0.013). Detailed information on PPDM-A complications is provided in Table 1, while clinical characteristics of patients in each cohort are outlined in Table 2.

**Table 1:** The detailed information regarding the complications associated with post-acute pancreatitis diabetes mellitus

PPDM-A Complications	Events; *n* (%) ^1^
diabetic ketoacidosis	21(27.27%)
hypoglycemia	11(14.29%)
microvascular complications	
nephropathy	7(9.09%)
neuropathy	18(23.38%)
retinopathy	7(9.09%)
diabetic foot	1(1.3%)
Infection	
pneumonia	20(25.97%)
abdominal infection	5(6.49%)
urinary tract infection.	8(10.39%)

Note: “^1^” the notation “n (%)” represents the number of cases (frequency). PPDM-A, post-acute pancreatitis diabetes mellitus

**Table 2:** The detailed clinical characteristics of the PPDM-A patients in the training, internal validation, and external validation cohorts

Characteristics	Institution 1 and Institution 2						Institution 3		
	Training cohort(*n* = 107)		P	Internal Validation cohort (*n* = 46)		P	External validation cohort (*n* = 73)		P
	complications (*n* = 34)	Non-complications (*n* = 73)		complications (*n* = 15)	Non-complications (*n* = 31)		complications (*n* = 28)	Non-complications (*n* = 45)	
Sex									
Male	25(73.53%)	46(63.01%)	0.284	12(80%)	24(77.42%)	0.842	22(78.57%)	39(86.67%)	0.364
Female	9(26.47%)	27(36.99%)		3(20%)	7(22.58%)		6(21.43%)	6(13.33%)	
Age(years)	44 ± 12	50 ± 16	0.032*	41 ± 12	51 ± 15	0.077	36 ± 10	43 ± 8	0.01*
Etiology									
Hypertriglyceridemia	16(47.06%)	20(27.4%)	0.134	9(60%)	9(29.03%)	0.128	18(64.28%)	31(68.89%)	0.489
Biliary	6(17.65%)	24(32.88%)		1(6.67%)	10(32.26%)		5(17.86%)	4(8.89%)	
Alcoholic	2(5.88%)	2(2.74%)		2(13.33%)	3(9.68%)		0	2(4.44%)	
Idiopathic	10(29.41%)	27(36.98%)		3(20%)	9(29.03%)		5(17.86%)	8(17.78%)	
CTSI	3(2 ~ 4)	4(3 ~ 6)	0.123	4(3 ~ 4)	4(2 ~ 6)	0.34	4(3 ~ 4)	4(3 ~ 4)	0.636
Disease severity									
Mild	11(32.35%)	12(16.44%)	0.006*	3(20%)	8(25.81%)	0.691	7(25%)	5(11.11%)	0.084
Moderate	18(52.94%)	59(80.82%)		12(80%)	22(70.97%)		19(67.86%)	29(64.45%)	
Severe	5(14.71%)	2(2.74%)		0	1(3.22%)		2(7.14%)	11(24.44%)	
EPIC	3(0 ~ 7)	5(3 ~ 7)	0.205	5(3 ~ 6)	5(2 ~ 6)	0.66	4(2.25 ~ 7)	5(3 ~ 6.5)	0.372
Hospital stay (days)	11(7 ~ 19)	13(9 ~ 21)	0.144	13(8 ~ 17)	13(8 ~ 20)	0.963	11.5(7.25 ~ 15.5)	13(9 ~ 18)	0.351
Follow-up time (months)	92(70.25 ~ 99.25)	78(63 ~ 112.5)	0.909	111(68 ~ 134)	80(79 ~ 111)	0.227	76(68 ~ 76)	78(77 ~ 79)	0.654
Pancreatic necrosis									
Yes	9(26.47%)	24(32.88%)	0.504	2(13.33%)	8(25.81%)	0.336	4(14.29%)	9(20%)	0.535
No	25(73.53%)	49(67.12%)		13(86.67%)	23(74.19%)		24(85.71%)	36(80%)	
recurrences									
Yes	18(52.94%)	53(72.6%)	0.045*	14(93.33%)	21(67.74%)	0.056	6(21.43%)	15(33.33%)	0.275
No	16(47.06%)	20(27.4%)		1(6.67%)	10(32.26%)		22(78.57%)	30(66.67%)	
Smoking									
Yes	18(52.94%)	33(45.21%)	0.456	6(40%)	15(48.39%)	0.592	13(46.43%)	21(46.67%)	0.984
No	16(47.06%)	40(54.79%)		9((60%))	16(51.61%)		15(53.57%)	24(53.33%)	
Drinking									
Yes	11(23.91%)	26(30.23%)	0.741	4(26.67%)	24(77.42%)	0.001*	13(46.43%)	25(55.56%)	0.448
No	23(76.09%)	47(69.77%)		11(73.33%)	7(22.58%)		15(53.57%)	20(44.44%)	
Fatty liver									
Yes	10(29.41%)	21(28.77%)	0.945	8(53.33%)	6(19.35%)	0.019*	19(67.86%)	31(68.89%)	0.926
No	24(70.79%)	52(71.23%)		7(46.67%)	25(80.65%)		9(32.14%)	14(31.11%)	
Ga+(mmol/L)	2.08 ± 0.34	2.12 ± 0.23	0.437	2.13 ± 0.29	2.09 ± 0.24	0.645	2.03 ± 0.29	1.99 ± 0.31	0.545
Cholesterol(mmol/L)	5.38(3.38 ~ 6.76)	4.35(3.35 ~ 5.78)	0.173	7.24(3.87 ~ 8.53)	3.92(3.28 ~ 6)	0.02*	7.53(4.69 ~ 9.12)	5.52(3.83 ~ 7.21)	0.013*
Triglyceride(mmol/L)	4.19(1.43 ~ 12.3)	2.06(1.26 ~ 4.79)	0.036*	7.59(2.89 ~ 16.68)	1.96(1.3 ~ 4.14)	0.01*	10.63(4.18 ~ 16.38)	3.95(1.98 ~ 7.44)	0.001*
Total bilirubin(umol/L)	17.2(13.68 ~ 23.25)	16.1(11.25 ~ 23.4)	0.454	19.3(13.3 ~ 28.9)	15.8(11.2 ~ 27.3)	0.639	14.9(9.93 ~ 23.95)	18.8(13.85 ~ 28.56)	0.114
Direct bilirubin(umol/L)	7.45(4.45 ~ 12.28)	6.5(3.9 ~ 10)	0.581	6.4(4.4 ~ 10.5)	8.6(3.7 ~ 12)	0.623	3.3(2.13 ~ 6.7)	3.3(2.01 ~ 6.2)	0.626
Creatinine(umol/L)	65.5(49.95 ~ 81.5)	63.2(48.7 ~ 75.75)	0.401	61.4(52.4 ~ 81.7)	64(54.5 ~ 76)	0.842	60.95(52.6 ~ 73.8)	61.1(50.86 ~ 79.1)	0.755
Urea(mmol/L)	4.84(4.17 ~ 6.19)	4.89(3.36 ~ 6.05)	0.506	5.36(3.81 ~ 6.3)	4.5(3.53 ~ 5.9)	0.631	3.85(2.56 ~ 5.21)	4.4(3.2 ~ 5.9)	0.19
Total protein(g/L)	68.4(61.2 ~ 77.73)	66.6(61.1 ~ 73.6)	0.478	71.2(63 ~ 80.9)	66.2(54 ~ 69)	0.013*	63.55(57.03 ~ 69.58)	62(55.35 ~ 67.05)	0.518
Albumin (g/L)	39.1(33 ~ 42.15)	37.9(32.25 ~ 41.1)	0.44	40.8(33 ~ 43.8)	37(32 ~ 40)	0.201	34(30.05 ~ 41.25)	36.2(31.8 ~ 38.55)	0.602
Hemoglobin(g/L)	140.44 ± 25.04	133.59 ± 26.79	0.212	148.93 ± 16.32	163.68 ± 183.33	0.759	135.12 ± 36.61	138.38 ± 28.29	0.671
Leukocyte(×10^− 9^/L)	10.63(7.86 ~ 16.39)	13.07(9.84 ~ 15.59)	0.246	13.28(10.02 ~ 17.09)	11.7(7.52 ~ 14.33)	0.228	11.37(8.5 ~ 13.73)	13.07(9.84 ~ 15.59)	0.905
ALT(IU/L)	34(20 ~ 61.25)	32(19.5 ~ 56.5)	0.761	28(16 ~ 41)	27(18 ~ 74)	0.379	22.25(12.48 ~ 35.1)	21.3(14.1 ~ 44.4)	0.427
AST(IU/L)	26(21 ~ 59.75)	31(24 ~ 48)	0.664	24(22 ~ 26)	27(22 ~ 55)	0.093	20.85(17.33 ~ 35)	22.5(17 ~ 40)	0.777
GGT(IU/L)	77(41.75 ~ 138.25)	75(36.5 ~ 157)	0.955	58(43 ~ 68)	51(36 ~ 127)	0.673	47(31.25 ~ 84.25)	95(45.5 ~ 145.45)	0.022*
ALP(IU/L)	79.5(61.75 ~ 91.75)	90(68 ~ 128)	0.102	68(63 ~ 75)	88(65 ~ 105)	0.073	91.5(75.75 ~ 128.5)	79(63 ~ 93)	0.031*

Note: PPDM-A, post-acute pancreatitis diabetes mellitus; CTSI, computed tomography severity index; EPIC, Extra-pancreatic Inflammation on CT; ALT, Alanine Transaminase; AST, Aspartate Transaminase, GGT, Gamma-Glutamyl transferase and ALP, Alkaline Phosphatase. *P < 0.05

### Radiomics, clinical and combined model construction, validation, and evaluation

Following the processes of data normalization, dimensionality reduction, feature selection, and classifier application, 1701 stable features were ultimately evaluated for model development. Through a comparative analysis of the AUCs on the validation dataset, six radiomics features and three clinical features emerged as the most predictive indicators. (Section S4 in the Supplementary Material for the detailed features information).

The radiomics model exhibited an AUC (with 95% CI) of 0.950 (0.906–0.993) in the training cohort, 0.888 (0.764–1.000) in the internal validation cohort, and 0.948 (0.880–1.000) in the external validation cohort.

The clinical model achieved an AUC (with 95% CI) of 0.707 (0.603–0.811) in the training cohort, 0.699 (0.529–0.868) in the internal validation cohort, and 0.713 (0.587–0.840) in the external validation cohort.

The combined model demonstrated an AUC (with 95% CI) of 0.954 (0.913–0.997) in the training cohort, 0.927 (0.856–0.998) in the internal validation cohort, and 0.933 (0.880–0.985) in the external validation cohort.

**Fig. 4:**
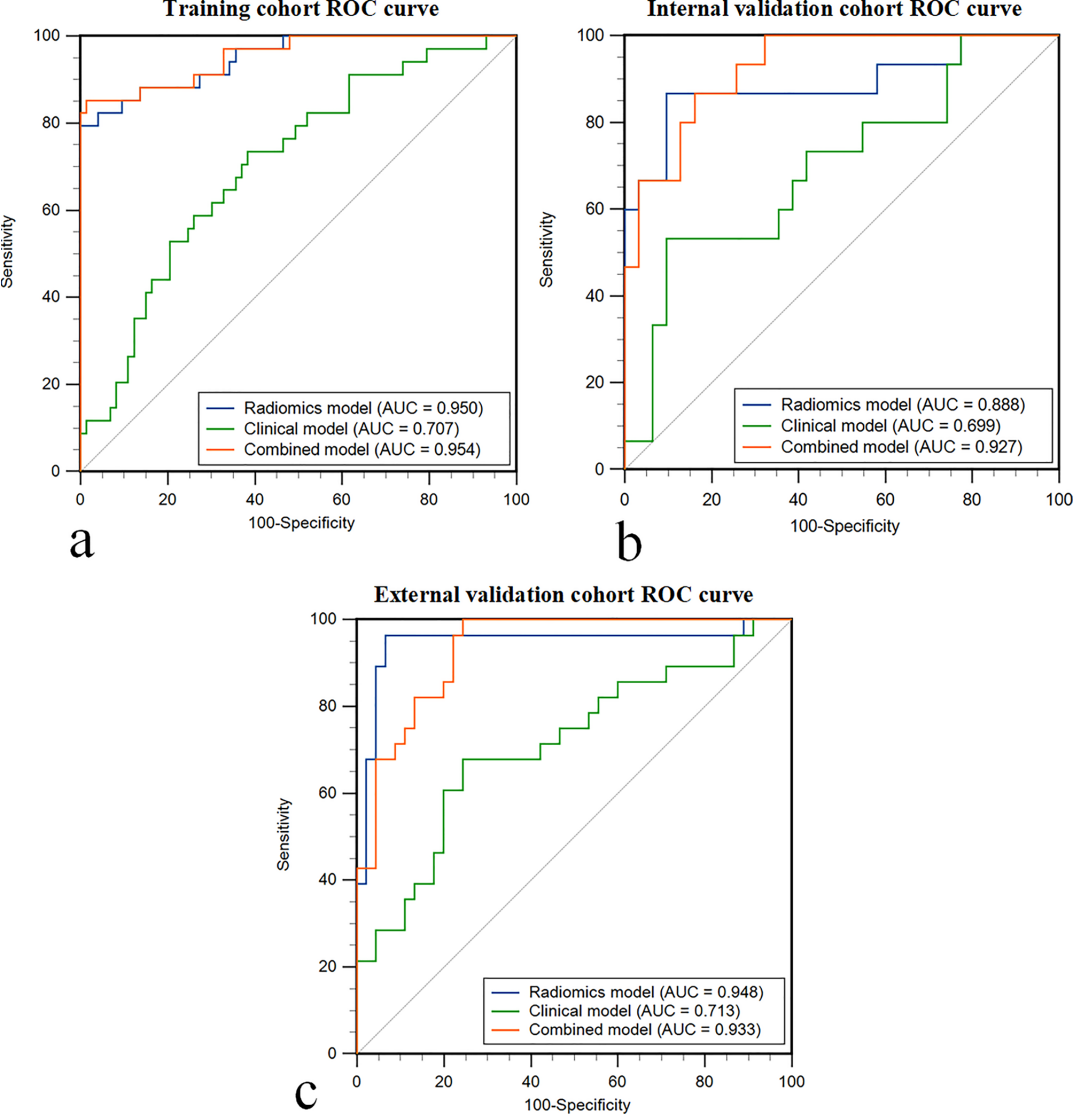
The Receiver Operating Characteristic Curve (ROC) of the radiomics model, clinical model, and combined model for predicting complications of post-acute pancreatitis diabetes mellitus. (**a**) ROC evaluation in training cohort. (**b**) ROC evaluation in internal validation cohort. (**c**) ROC evaluation in external validation cohort

A comparison of the AUCs among the radiomics, clinical, and combined models revealed that the radiomics model significantly outperformed the clinical model in the external validation cohort (0.948 vs. 0.713, *p* = 0.002). However, no significant difference was observed between the combined and radiomics models (0.933 vs. 0.948, *p* = 0.638). Figure 4 illustrates the Receiver Operating Characteristic Curve evaluation of the models, while Table 3 provides detailed information on the models.

**Table 3:** The performance of the radiomics model, clinical model, and combined model in the training cohort, the internal validation cohort and the external validation cohort

	AUC(95% CI)			Accuracy	Sensitivity	Specificity	PPV	NPV
Training Cohort								
Clinical	0.707(0.603,0.811)			0.654	0.735(25/34)	0.616(45/73)	0.472(25/53)	0.833(45/54)
Radiomics	0.950(0.906,0.993)			0.935	0.764(26/34)	1.0(73/73)	1.0(26/26)	0.901(73/81)
Combined	0.954(0.913,0.997)			0.944	0.853(29/34)	0.986(72/73)	0.967(29/30)	0.935(72/78)
Internal Validation Cohort								
Clinical	0.699(0.529,0.868)			0.783	0.533(8/15)	0.903(28/31)	0.727(8/11)	0.8(28/35)
Radiomics	0.888(0.764,1.0)			0.891	0.866(13/15)	0.903(28/31)	0.812(13/16)	0.933(28/30)
Combined	0.927(0.856,0.998)			0.848	0.867(13/15)	0.839(26/31)	0.722(13/18)	0.929(26/28)
External Validation Cohort								
Clinical	0.713(0.587,0.84)			0.726	0.679(19/28)	0.756(34/45)	0.633(19/30)	0.790(34/43)
Radiomics	0.948(0.880,1.0)			0.945	0.964(27/28)	0.933(42/45)	0.9(27/30)	0.977(42/43)
Combined	0.933(0.880,0.985)			0.849	1.0(28/28)	0.756(34/45)	0.718(28/39)	1.0(34/34)

Note: PPV, positive predictive value; NPV, negative predictive value; AUC, area under the receiver operating characteristic curve; CI, confidence interval. The brackets in the table show the numerator and denominator of the sensitivity, specificity, PPV and NPV

### Model interpretability with SHAP

**Fig. 5:**
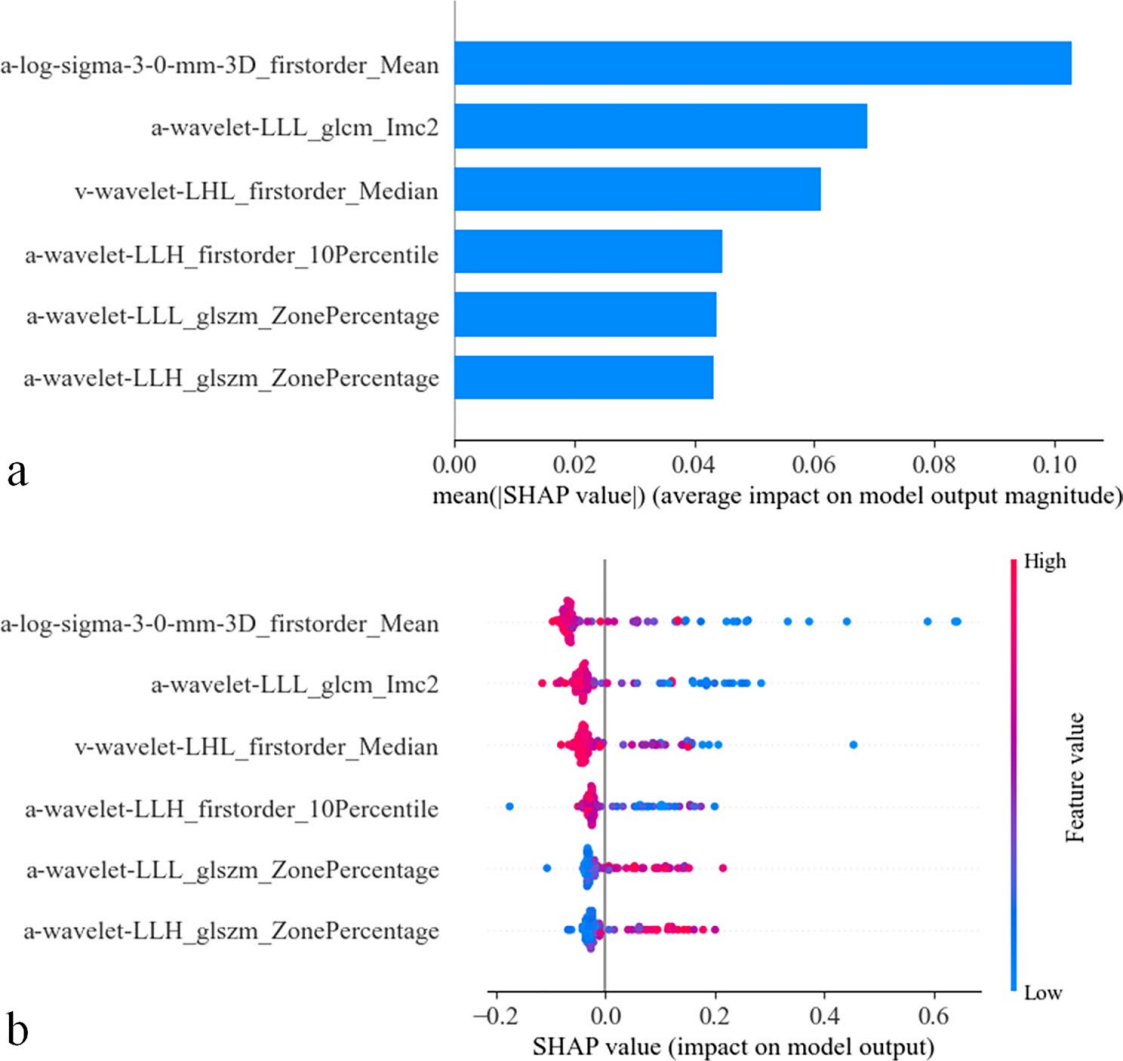
The SHapley Additive exPlanations (SHAP) plots for the radiomics model. (**a**) The SHAP feature importance plot rank features by their significance in the model’s predictions, showing which features contribute most. (**b**) The SHAP summary plot offers an overview of feature impacts and interactions. Features arranged in descending order of importance. Points on the plot indicate SHAP values, colored from blue (low) to red (high). Points right of the Y-axis suggest a higher likelihood of PPDM-A complications, while those left indicate a lower likelihood

SHAP values were computed for all the selected radiomic features. Figure 5a displays a plot of SHAP feature importance, ranking the features in descending order of their significance. The top features had a greater influence on the model and higher predictive power compared to the bottom ones. Notably, the first-order mean of the arterial-phase CECT images emerged as the most impactful feature on the prediction outcomes.

Figure 5b presents the SHAP summary plot, which illustrates the impact of features on the radiomics model’s decisions and the interactions between them. The features are also listed in order of importance from top to bottom. Each point on the plot represents the SHAP value of a patient’s feature, ranging from low (blue) to high (red). Points to the right of the Y-axis indicate an increased likelihood of PPDM-A complications, while those to the left suggest a decreased likelihood. Taking “first-order mean” as an example: Positioned on the Y-axis right, this feature’s bluer color (smaller value) correlates with higher patient complication risk.

**Fig. 6:**
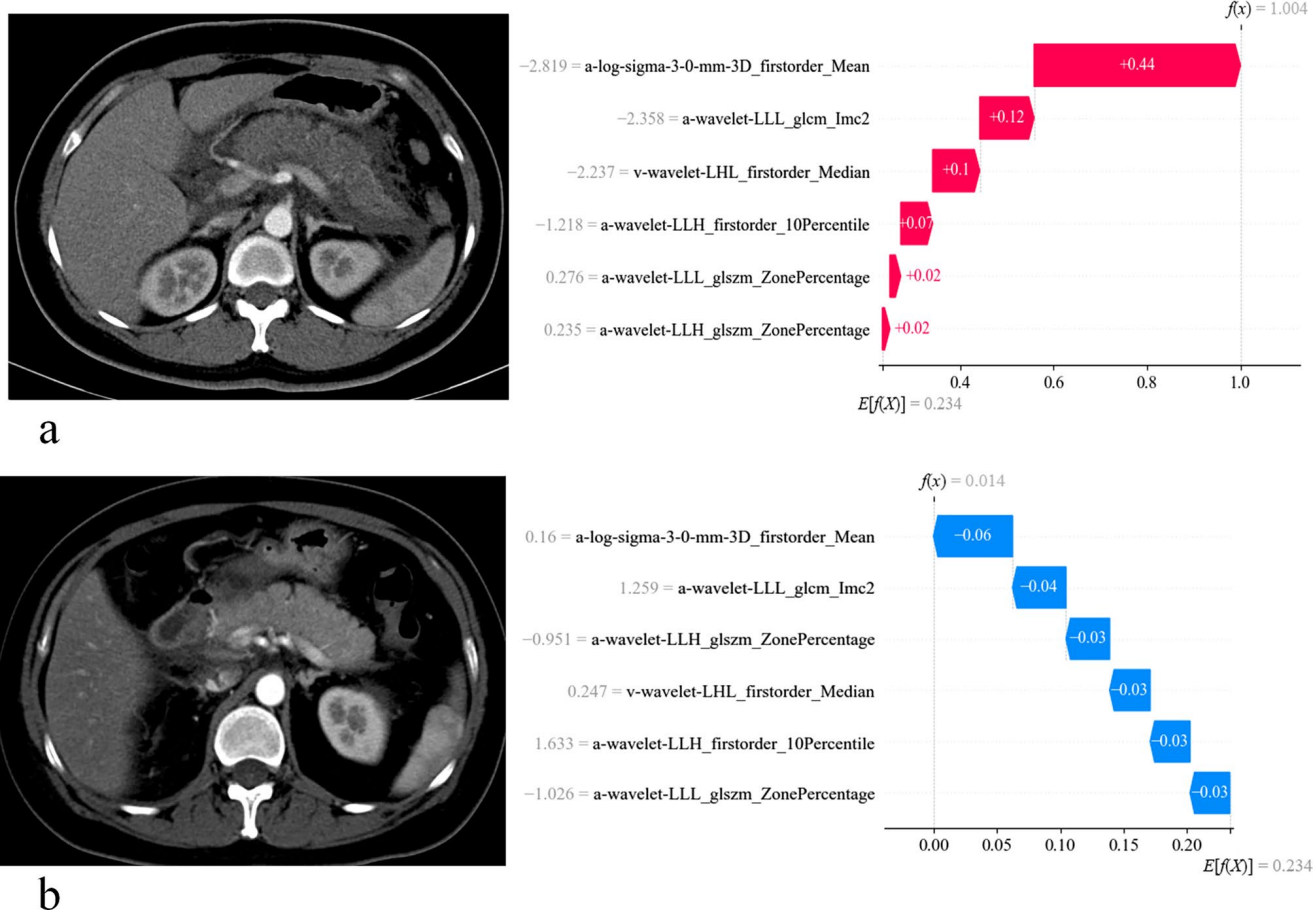
The SHapley Additive exPlanations (SHAP) waterfall plots illustrated the interpretability of the radiomics model at an individual level. Specifically, red bars signify an increase in predictive value, while blue bars denote a decrease. When considering the cumulative effect of all features, a definitive predictive value is established. If this value falls below the threshold of 0.234 (the base value), the prognosis indicates the absence of complications. For illustrative purposes, patients (**a**) and (**b**) were arbitrarily chosen from the radiomics model, representing cases with and without complications, respectively. **a**: The total of values on the red bars was 1.004, which exceeded the threshold (0.234) and thus indicated the presence of complications in the patient. **b**: The total of values on the blue bars was 0.014, which was below the threshold (0.234) and thus indicated the absence of complications in the patient

To demonstrate individual sample predictions, we randomly selected two patients and created SHAP waterfall plots (Fig. 6). These plots visualize the SHAP value of each feature as a bar that either increases or decreases the prognosis. Each prediction starts with a base value (0.234), which represents the average SHAP value of all predictions. The length of the bar indicates the contribution of a particular feature to the SHAP value, with red bars signifying an increased predictive value and blue bars signifying a decreased predictive value. Figure 6a: The total of values on the red bars was 1.004, which exceeded the base value(0.234)and thus indicated the presence of complications in the patient. Figure 6b: The total of values on the blue bars was 0.014, which was below the base value (0.234) and thus indicated the absence of complications in the patient.

**Fig. 7:**
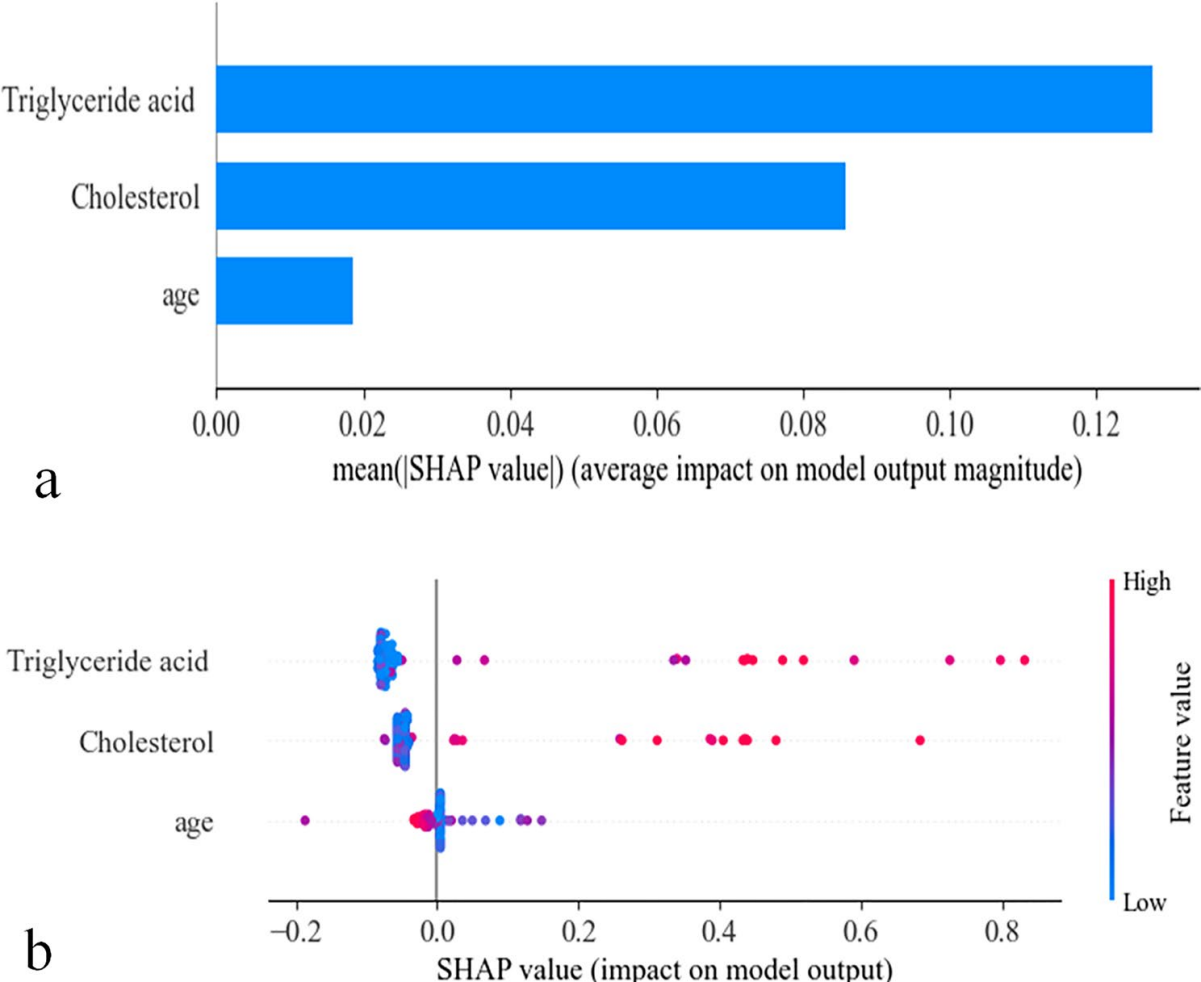
The SHapley Additive exPlanations (SHAP) plots for the clinic model. (**a**) The SHAP feature importance plot. (**b**) The SHAP summary plot. Among the features, triglyceride acid exerted the largest average impact on the model output, followed by cholesterol, while age had the smallest impact

In this study, the clinical model was constructed using three clinical indicators: triglyceride acid, cholesterol, and age. As shown in Fig. 7, triglyceride acid had the largest average impact on the model output, followed by cholesterol, while age had the smallest impact.

## Discussion

In this study, we developed and validated a quantitative radiomics model using CECT images to predict PPDM-A complications non-invasively and personally. The model performed in the external validation cohort with an AUC of 0.948. Furthermore, interpretation of the model using the SHAP technique revealed that the radiomics features derived from CECT images were closely linked to the prognosis of PPDM-A.

In this study, we observed an incidence of 27.27% for diabetic ketoacidosis and 14.29% for hypoglycemia among patients with PPDM-A complications. Previous reports have indicated that patients with PPDM-A exhibit a tendency for higher risks of both diabetic ketoacidosis and hypoglycemia compared to those with T2DM [9]. Furthermore, over a period of five years, patients with PPDM-A experienced a significantly higher incidence of poor glycemic control (HbA1c ≥ 7%) compared to T2DM patients (61.9% vs. 46.3%), along with elevated HbA1c levels (8.3% vs. 7.9%) [7]. Additionally, our study found that the incidence of diabetic microvascular damage in PPDM-A complication group was 42.86%. Specifically, the incidence rates for diabetic nephropathy, retinopathy, neuropathy, and diabetic foot were 9.09%, 9.09%, 23.38%, and 1.3%, respectively. These findings suggest that diabetic microvascular damage is a common complication in patients with PPDM-A. Moreover, the incidence of infections was 42.85%, including pneumonia, urinary tract infections, and abdominal infections. This high incidence may be attributed to the fact that during AP attacks, the incidence of extra-pancreatic infectious complications can reach as high as 32% [26]. Additionally, poor glycemic control in PPDM-A patients further increases the risk of developing diabetic infections [27].

In our study, we found significantly elevated triglyceride levels in the PPDM-A complications group compared to the non-complications group among all cohorts. This finding consistent with previous research indicating that inflammation-induced lipolysis notably increases serum triglyceride levels in PPDM-A patients [28]. Elevated triglycerides may exacerbate insulin resistance, leading to diabetes and its complications [29]. Additionally, cholesterol levels were significantly higher in complications group in both internal and external validation cohorts, with a trend towards higher levels in training cohort. Abnormal cholesterol metabolism is linked to diabetic microvascular complications, particularly diabetic retinopathy [30, 31]. Furthermore, patients in the complications group tended to be younger, suggesting that younger age at AP onset may increase the risk of future PPDM-A complications. Significant differences in recurrence rates, disease severity, drinking status, fatty liver, and total protein were observed only in specific cohorts, possibly due to selection bias from small sample size. Despite feature selection, the clinical model’s predictive accuracy for PPDM-A complications was low (0.726).

Compared to the clinical model, our radiomics model showed significant advantages in predicting PPDM-A complications. Based on CECT images from the first AP episode, our findings suggest that some patients are already at high risk for adverse outcomes. Chronic inflammation caused by elevated pro-inflammatory cytokines during AP attacks is a key factor in PPDM-A development [32]. These cytokines can cause early retinal neuron cell death, promoting diabetic retinopathy [33]. Pro-inflammatory cytokines also can stimulate lipolysis, redistribute body fat, deposit fat in pancreas, damage islets and disrupt insulin secretion, leading to PPDM-A and complications [34–36]. Pancreatic microcirculation impairment during AP may also damage Langerhans islet cells and impair insulin secretion [37, 38]. Therefore, changes in the pancreatic microenvironment during the first AP episode may cause PPDM-A complications. Radiomics can detect early, non-visual microenvironmental changes, enabling accurate predictions of PPDM-A complications.

Although radiomics models are widespread and powerful, their clinical use depends on interpretability. The SHAP technique provides both global and local interpretability, helping understand how features influence predictions. The SHAP summary plot visually shows feature importance, with wider dots indicating more significant features and colors (blue to red) showing impact range. In our study, clinicians could easily see how features like the first-order Mean affected assessments. The first-order Mean, the strongest non-semantic feature, reflects image brightness [16, 39]. Patients with PPDM-A complications had lower first-order Mean values, suggesting darker images due to increased fat or poor vascular supply. This can damage Langerhans islet cells, impair insulin secretion, and disrupt glucose metabolism, leading to diabetes and complications [35–37].

After understanding feature impacts on the radiomics model, clinicians use a SHAP waterfall plot for individual patient assessments. By comparing a patient’s SHAP value to the base value, they can categorize them into the PPDM-A complications group if the SHAP value exceeds the base. The bar colors (red for increased predictive value) and lengths (indicating contribution degree) help discern feature impacts. As shown in Fig. 6a, both a-log-sigma-3-0-mm-3D firstorder Mean and a-wavelet-LLL glcm lmc2 contribute positively, with a-log-sigma-3-0-mm-3D firstorder Mean having a greater influence.

Previous studies on PPDM-A prediction can be categorized into three types: clinical factor-based models, radiomics models, and combined models. Clinical models (e.g., the PERSEUS score, nomograms developed by Soo et al. and Zhang et al.) rely on limited clinical indicators (e.g., recurrence times, follow-up duration, and basic metabolic parameters) [12, 14]. Their predictive accuracy is relatively low due to the weak specificity of clinical features and failure to capture subtle pancreatic structural changes. Zhong et al. constructed a radiomics nomogram based on unenhanced CT images, but it achieved only a moderate AUC of 0.787 [18], constrained by a single-center design, small sample size, and neglect of valuable contrast-phase information. Our previous radiomics model for PPDM-A incidence prediction yielded an external validation AUC of 0.857 [17], while the current model—optimized for complication prediction by integrating arterial- and venous-phase contrast-enhanced CT features—achieves an AUC of 0.948, demonstrating stronger discriminative power for adverse outcomes. Compared with existing tools, our model has several advantages. First, it integrates multi-phase contrast-enhanced CT features from three tertiary hospitals, avoiding the limitations of single-center data and ensuring better generalizability. Second, with an external validation AUC of 0.948, it outperforms clinical models [12, 14], single-phase radiomics models (AUC = 0.787) [18], and even our previous incidence-prediction radiomics model (AUC = 0.857) [17], particularly in identifying high-risk patients for complications. Third, integrating SHAP technology addresses the “black box” issue of traditional radiomics models, visualizing feature contributions and individual risk assessment processes. This interpretability, combined with non-invasive imaging data acquisition, makes our model more suitable for routine clinical application and more credible than complex clinical scoring systems or uninterpretable machine learning models.

Our study has some limitations. First, the retrospective design is prone to selection and information biases. Data were retrieved from medical records and imaging archives across three centers, with inconsistencies in documentation completeness and standardization that may compromise data accuracy. Second, the study population is relatively homogeneous, comprising patients from tertiary hospitals in Chongqing with similar baseline characteristics, which may limit the generalizability of the findings. Third, despite the relatively short follow-up for chronic complications, early onset is common in 30% of newly diagnosed T2DM patients, with significant complications potentially arising within 4.1 years [40, 41]. Our study’s follow-up period, spanning approximately 7.5 years from the initial AP episode to complications onset, is thus deemed adequate for evaluating chronic complications, given our previous finding of a roughly 2.4-year interval between the first AP episode and PPDM-A emergence [17]. Lastly,

the relatively small sample size may compromise the model’s generalization ability by failing to fully capture clinical and radiomic heterogeneity of PPDM-A patients and elevate overfitting risk. While we mitigated these issues via 5-fold cross-validation, overfitted model exclusion, and external validation (AUC = 0.948), subgroup analyses were not performed due to limited data, with our focus restricted to microvascular complications, infections, diabetic ketosis, and hypoglycemia. Notably, we have recruited PPDM-A patients from three tertiary centers over the past decade and will expand the cohort to enable subgroup analyses in future studies.

In Conclusion, the CECT‑based radiomic model showed favorable prognostic performance for PPDM‑A, and SHAP analysis improved model interpretability to support its clinical application.

## Supplementary Information

Below is the link to the electronic supplementary material.

Supplementary Material 1

Supplementary Material 2

## Data Availability

Due to data sensitivity and the requirements stipulated by the Institutional Review Boards (IRBs) of The First Affiliated Hospital of Chongqing Medical University, The Second Affiliated Hospital of Chongqing Medical University, and Southwest Hospital, the data supporting this study’s findings are not publicly accessible. These data are securely stored at the aforementioned hospitals. The data that support the findings of this study are available from the corresponding author, Hua Yang, upon reasonable request. Correspondence and data requests should be addressed to Hua Yang at: 13527547568@163.com.

## Abbreviations

CECT: Contrast enhanced computed tomography
PPDM: A Post-acute pancreatitis diabetes mellitus
SHAP: Shapley Additive exPlanations
FAE: FeAture Explorer
AUC: The area under the receiver operating characteristic curve
AP: Acute pancreatitis
T2DM: Type 2 diabetes mellitus
CTSI: CT severity index
EPIC: Extra-pancreatic inflammation on CT
ALT: Alanine Transaminase
AST: Aspartate Transaminase
GGT: Gamma-Glutamyl transferase
ALP: Alkaline Phosphatase
ICCs: Intraclass correlation coefficients
PCC: Pearson correlation coefficients
ANOVA: Analysis of variance
RFE: Recursive feature elimination
