# Supplementary material for: An interpretable radiomics model based on contrast‑enhanced pancreatic computed tomography for predicting the prognosis of post-acute pancreatitis diabetes mellitus
Source: BMC Med Imaging. 2026 Mar 13;26:201. doi: 10.1186/s12880-026-02258-7 (PMC13097733; doi:10.1186/s12880-026-02258-7)
Supplement: Supplementary file 1 — Supplementary Material 1 [file 12880_2026_2258_MOESM1_ESM.pptx]

## Slide 1
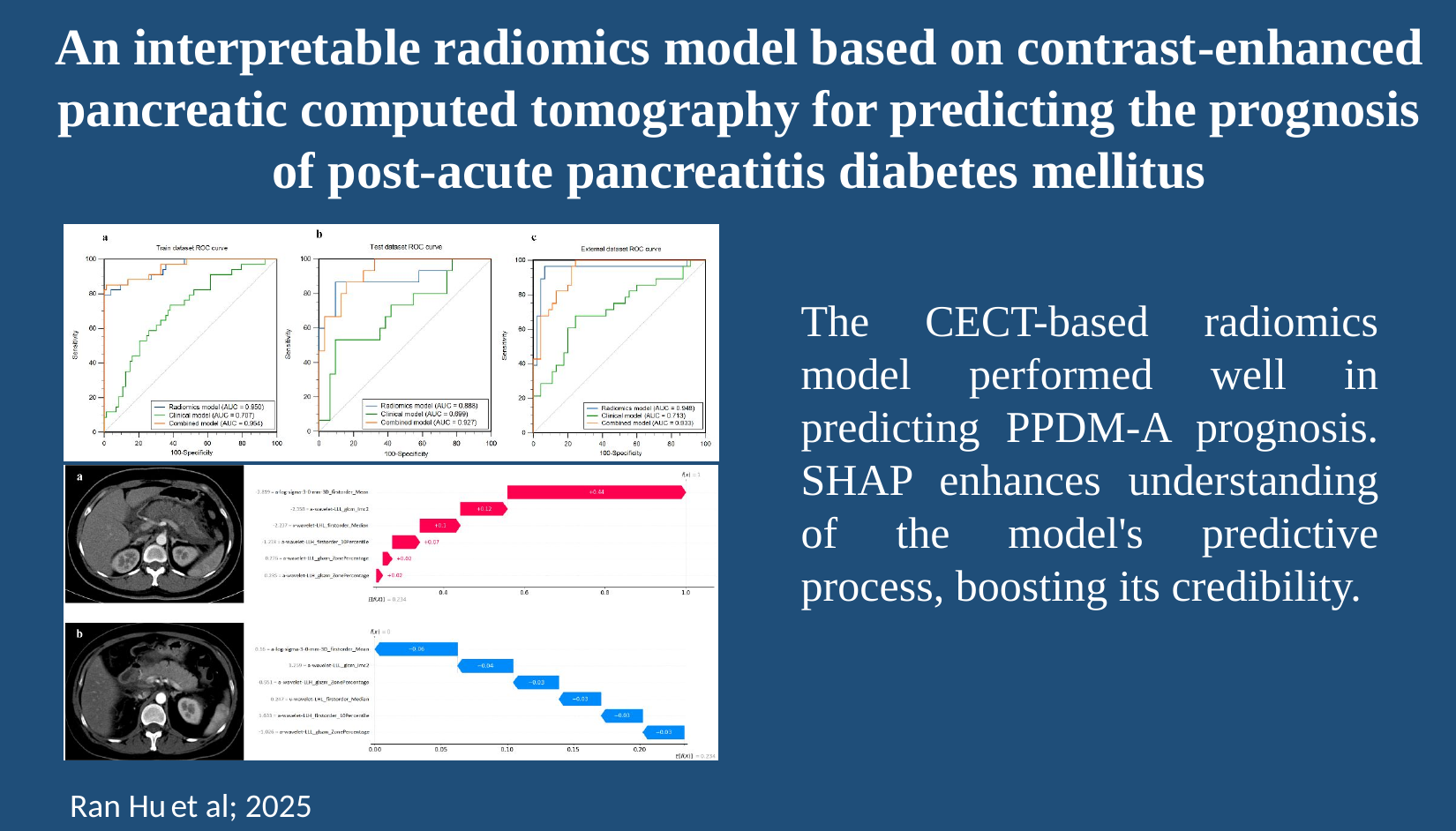

An interpretable radiomics model based on contrast‑enhanced pancreatic computed tomography for predicting the prognosis of post-acute pancreatitis diabetes mellitus
The CECT-based radiomics model performed well in predicting PPDM-A prognosis. SHAP enhances understanding of the model's predictive process, boosting its credibility.
Ran Hu et al; 2025
