# Supplementary material for: An interpretable radiomics model based on contrast‑enhanced pancreatic computed tomography for predicting the prognosis of post-acute pancreatitis diabetes mellitus
Source: BMC Med Imaging. 2026 Mar 13;26:201. doi: 10.1186/s12880-026-02258-7 (PMC13097733; doi:10.1186/s12880-026-02258-7)
Supplement: Supplementary file 2 — Supplementary Material 2 [file 12880_2026_2258_MOESM2_ESM.docx]

**Supplementary Material**

**S1. The diagnostic criteria for acute pancreatitis and diabetes.**

**S1.1** According to the 2012 Atlanta consensus criteria [1], AP was diagnosed based on the identification of at least two of the following three signs: (a) the presence of typical abdominal pain; (b) elevated serum amylase or lipase levels at least three times greater than the upper limit of normal; and (c) characteristic imaging findings of AP.

**S1.2** Diabetes was diagnosed by recording at least one of the following [2]: (a) fasting plasma glucose levels ≥ 126mg/dl (7.0 mmol/L); (b) plasma glucose ≥11.1 mmol/L two hours after a 75 g oral glucose load; (c) random blood glucose≥ 200mg/dl (11.1mmol/L) accompanied by diabetes symptoms; and (d) Glycosylated hemoglobin ≥ 6.5% (48 mmol/dl).

**S2. Diagnostic criteria for diabetic complications.**

**S2.1** The criteria diagnosis for diabetic ketoacidosis included: serum glucose over 250 mg/dL, pH less than 7.3, bicarbonate level less than 18 meq/L, and elevated serum ketones (beta-hydroxybutyrate);

**S2.2** Hypoglycemia was defined as any incident of low blood sugar during the follow-up period that required hospital admission.

**S2.3** Microvascular complications arising after diabetes diagnosis encompass nephropathy, neuropathy, retinopathy, and diabetic foot. These were defined as follows:

(1) Neuropathy was confirmed through the presence of sensorimotor and autonomic neuropathic symptoms, typical sensorimotor deficits identified during a physical examination, or a formal diagnosis provided by a specialist physician.

(2) Nephropathy was characterized by elevated creatinine levels (exceeding 90 μmol/L for women and 100 μmol/L for men), the presence of proteinuria (indicated by an albumin-to-creatinine ratio of ≥3 mg/mmol in a spot urine sample or albumin levels exceeding 20 mg/L in a 24-hour urine specimen), or a formal diagnosis made by a specialist physician.

(3) Retinopathy, regardless of stage, was documented during visits to an ophthalmology outpatient clinic.

(4) Diabetic foot was confirmed through the presence of foot ulcers or a formal diagnosis provided by a specialist physician.

**S2.4** Infection, defined as the emergence of bacterial infections that necessitate either inpatient or outpatient antibiotic therapy and occur between the time of diabetes diagnosis and the last contact, encompasses pneumonia, abdominal infections, and urinary tract infections.

**S3.** CECT image collection

All patients underwent CECT upon abdominal imaging using one of the six following multidetector row CT systems: Aquilion ONE (Toshiba, Tokyo, Japan), Ingenuity CT (Philips Medical System), Optima CT 660(General Electric Company), Somatom Definition AS+, Somatom Force and Somatom Definition Flash (Siemens Healthineers). The multidetector row CT systems and detailed image collection information is provided in Supplementary table 1. For the first five CT scanners, after a routine nonenhanced scan, arterial- and portal venous-phase CECT scans were performed after 25–30 s and 48–50 s of delay following the intravenous administration of iodinated contrast material (Ultravist 370, Bayer Schering Pharma) at 1.5 mL/kg at a rate of 3 mL/s using a pump injector. For Somatom Definition Flash, an automatic exposure control system (Care Dose 4D; Siemens Medical Solutions) was used when performing scanning. And after a routine nonenhanced scan, arterial- and portal venous-phase CECT scans were performed after 25 s and 40 s of delay following the intravenous administration of iodinated contrast material (Ultravist 370, Bayer Schering Pharma) at 1.5 mL/kg at a rate of 3.5–5 mL/s using a pump injector.

**S4.** **The detail information of selected radiomics and clinical features**

**S4.1. Radiomics features of arterial phase**

- Log-sigma-3-0-mm-3D_firstorder_Mean
- wavelet-LLH_firstorder_10Percentile
- wavelet-LLH_glszm_ZonePercentile
- wavelet-LLL_glcm_Imc2
- wavelet-LLL_glszm _ZonePercentage

**S4.2. Radiomics features of venous phase**

- wavelet-LHL_firstorder_Median

**S4.3. clinical features**

- age
- cholesterol
- triglyceride acid

**Reference**

[1] Banks PA, Bollen TL, Dervenis C et al (2013) Classification of acute pancreatitis–2012: revision of the Atlanta classification and definitions by international consensus. Gut 62:102–111

[2] American Diabetes Association Professional Practice Committee. 2 (2024) Diagnosis and Classification of Diabetes: Standards of Care in Diabetes-2024. Diabetes Care 47:S20-S42

**Supplementary table 1** The multidetector row CT systems and detailed image collection information

| CT scanners | tube current（mA） | tube voltage（kVp） | slice thickness（mm） | pitch | collimation（mm） | Rotation time（s） |
| --- | --- | --- | --- | --- | --- | --- |
| Aquilion ONE | 250 | 120 | 5 | 0.87 | 320×0.5 | 0.75 |
| Ingenuity CT | 250 | 120 | 5 | 1.015 | Auto | 1 |
| Optima CT660 (GE) | 400 | 120 | 5 | 0.984 | 64×0.625 | 0.5 |
| SOMATOM Definition AS+ | 250 | 120 | 5 | 0.6 | 128×0.6 | 0.5 |
| SOMATOM Force | Auto | 120 | 5 | 0.6 | 128×0.6 | 0.5 |
| SOMATOM Definition Flash | 318 | 100 | 5 | 0.8 | 128×0.6 | 0.5 |
